# Neural stem cell-loaded biohybrid hydrogel improves cochlear implants by electrode-neural coupling and neural regeneration

**DOI:** 10.7150/thno.120515

**Published:** 2026-01-01

**Authors:** Menghui Liao, Xin Zhou, Hao Wei, Yanru Qi, Pan Feng, Xin Gao, Yangnan Hu, Yuyang Qiu, Yusong Wang, Hongbo Yang, Zhonghong Zhang, Zhongze Gu, Renjie Chai

**Affiliations:** 1 State Key Laboratory of Digital Medical Engineering, Department of Otolaryngology Head and Neck Surgery, Zhongda Hospital, School of Life Sciences and Technology, Advanced Institute for Life and Health, Jiangsu Province High-Tech Key Laboratory for Bio-Medical Research, Southeast University, Nanjing, 210096, China.; 2 School of Medical Engineering, Affiliated Zhuhai People's Hospital, Beijing Institute of Technology, Zhuhai, 519088, China.; 3 Co-innovation Center of Neuroregeneration, Nantong University, Nantong, 226001, China.; 4 State Key Laboratory of Digital Medical Engineering, School of Biological Science and Medical Engineering, Southeast University, Nanjing, 211189, China.; 5 Department of Otolaryngology Head and Neck Surgery, Nanjing Drum Tower Hospital, Affiliated Hospital of Medical School, Nanjing University, Jiangsu Provincial Key Medical Discipline (Laboratory), Nanjing, 210009, China.; 6 Research Institute of Otolaryngology, Nanjing, 210009, China.; 7 Obstetrics and Gynecology Hospital, Institute of Reproduction and Development, Fudan University, Shanghai, 200032, China.; 8 Department of Ophthalmology, Zhongda Hospital, Southeast University, Nanjing, 210009, China.; 9 Department of Neurology, Aerospace Center Hospital, School of Life Science, Beijing Institute of Technology, Beijing, 100081, China.; 10 Department of Otolaryngology Head and Neck Surgery, Sichuan Provincial People's Hospital, University of Electronic Science and Technology of China, Chengdu, 610072, China.; 11 Southeast University Shenzhen Research Institute, Shenzhen, 518063, China.

**Keywords:** neural stem cell, PEDOT:PSS, collagen hydrogel, cochlear implants, sensorineural hearing loss

## Abstract

**Background:** Contemporary cochlear implants (CIs) face unresolved dual challenges: biomechanical-electrochemical mismatch at the electrode-tissue interface and progressive spiral ganglion neuron (SGN) degeneration, severely limiting long-term auditory restoration. Integrating regenerative medicine with bioelectronic engineering offers promise to overcome these bottlenecks.

**Methods:** A biohybrid neural interface was developed by embedding neural stem cells (NSCs) in photopolymerized poly(3,4-ethylenedioxythiophene):poly(styrenesulfonate) (PEDOT:PSS)/collagen hydrogel. Physicochemical properties were characterized via rheometry, electron microscopy, and electrochemical impedance spectroscopy. *In vitro* NSC responses (proliferation/differentiation) were quantified with EdU/Tuj1 assays. Therapeutic efficacy was evaluated in guinea pigs with ouabain-induced auditory neuropathy using auditory brainstem response (ABR) thresholds and immunohistochemical SGN quantification, comparing CI-alone versus NSC-hydrogel-CI groups.

**Results:** The photopolymerized PEDOT:PSS/collagen hydrogel demonstrated cochlear tissue-matched viscoelastic properties (storage modulus: 8.7-12.4 kPa) with injectable sol-gel transition capability, while exhibiting enhanced bioelectronic coupling through high electrical conductivity (1.3 ± 0.1 S/m) and 97.7% reduction in charge transfer resistance. This electroactive microenvironment significantly promoted NSC proliferation (+51.6%) and neuronal differentiation (+76.4%) *in vitro*, effects further amplified by CI stimulation to achieve +71.5% proliferation and +23.4% neuronal differentiation. *In vivo* evaluation using ouabain-induced auditory neuropathy guinea pigs revealed substantial functional recovery, with ABR threshold improvements of 18.8-28.8 dB across 4-12 kHz frequencies by post-operative day 14, correlating with significant SGN regeneration in the apical turn (+11.14 cells/0.01 mm²), whereas CI-alone controls exhibited negligible recovery.

**Conclusions:** This NSC-laden conductive hydrogel establishes a self-reinforcing therapeutic paradigm that simultaneously resolves electrode-tissue mismatch through optimized bioelectronic interfacing and reverses neurodegeneration via stem cell-mediated SGN regeneration. The dual-function platform pioneers active neural repair for next-generation neuroprosthetics.

## Introduction

The development of high-fidelity bioelectronic interfaces remains a critical challenge in neural prosthetics, particularly for cochlear implants (CIs) 1, 2, which represent the most clinically successful neurotechnology for restoring auditory function in sensorineural hearing loss (SNHL) 3-5. Contemporary CI systems bypass damaged cochlear hair cells by electrically stimulating residual spiral ganglion neurons (SGNs), yet their long-term efficacy is constrained by two limitations 6, 7. The first challenge arises from biomechanical-electrochemical mismatch: Conventional Pt-Ir electrodes (Young's modulus: GPa range) exhibit a five-order mechanical mismatch with cochlear soft tissues (kPa range) 8, 9, inducing chronic shear stresses during micromotion that activate pro-inflammatory mechanotransduction pathways 10-13. Electrically, these metallic interfaces demonstrate limited charge injection capacity, typically capped at approximately 0.1 mC/cm^2^. To achieve effective neural activation, elevated stimulation currents are required, which inadvertently induce excitotoxic SGN apoptosis through calcium-mediated caspase activation 1, 14, 15. The second limitation stems from progressive SGN degeneration: Histopathological studies reveal that CI candidates already suffer an average pre-implantation SGN loss of 58.8%, a condition further exacerbated post-implantation due to sustained inflammation and oxidative stress from reactive oxygen species accumulation 16-19. This neuronal depletion creates a vicious cycle: diminished SGN populations necessitate higher stimulation currents, which in turn accelerate neuronal apoptosis, leading to progressive degradation of CI performance over time.

Recent advancements in conductive hydrogels engineering offer promising solutions to address biomechanical-electrochemical mismatch. Through strategic polymer matrix engineering, these materials simultaneously achieve dual objectives: mechanical compliance matching the kPa-range viscoelasticity of cochlear tissues, and enhanced electrochemical performance with charge injection capacities >1 mC/cm^2^ 20-22. Poly(3,4-ethylenedioxythiophene):poly(styrenesulfonate) (PEDOT:PSS)-incorporated hydrogels exemplify this approach, enabling hybrid ionic-electronic conduction superior to metallic conductors 23-26. Their percolated conductive networks reduce electrode-tissue impedance while their porous architectures facilitate nutrient diffusion and cell-material interactions. However, prevailing research predominantly prioritizes signal transduction optimization, overlooking a fundamental biological reality: when the target neuronal population (SGNs) is severely depleted, even an ideal electrode cannot effectively restore neural encoding functions. This critical oversight—the failure to integrate neural regeneration imperatives—represents a major barrier to achieving sustainable CI efficacy.

**Figure 1:**
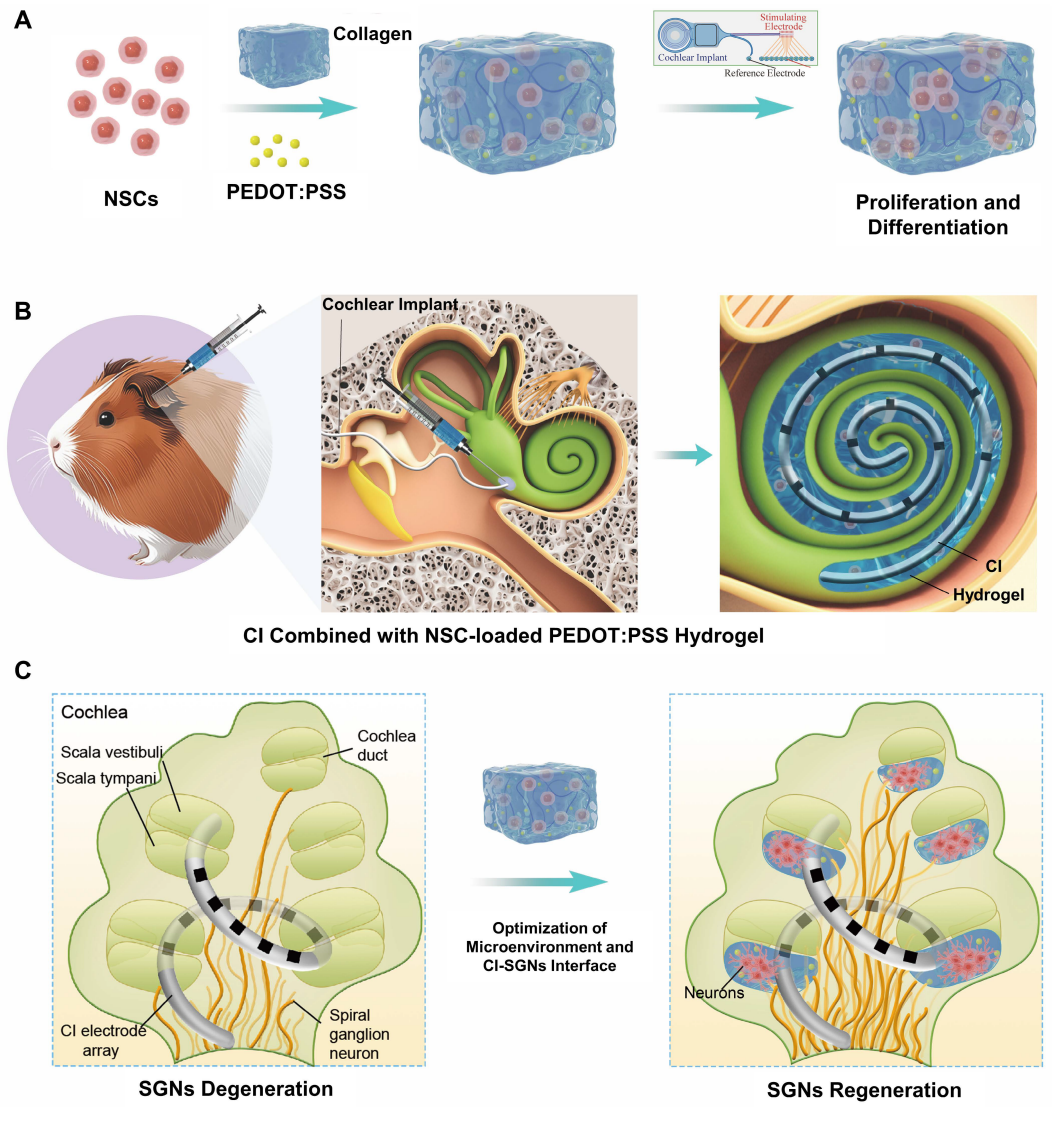
Schematic illustration of NSC-loaded PEDOT:PSS/collagen hydrogel enhancing CI performance through electrode-neural coupling and neural regeneration. (A) PEDOT:PSS/collagen hydrogel provides a compatible 3D microenvironment for NSCs to modulate NSC proliferation and differentiation. (B) Integration of NSC-loaded hydrogel with CI for treating neuropathic hearing loss. (C) Combined therapeutic approach optimizes the microenvironment and bioelectronic interface conductivity to facilitate auditory function restoration.

To overcome these intertwined challenges, we engineered a biohybrid hydrogel system integrating a PEDOT:PSS/collagen matrix hydrogel with neural stem cells (NSCs) to enhance CI efficacy (**Figure 1**). This tripartite design synchronizes interface optimization with neural reconstruction. The collagen matrix enables temperature-responsive injectability via physiological sol-gel transition, ensuring precise intraoperative delivery and conformal filling of electrode-tissue interfaces to mitigate micromotion-induced inflammation 27. The percolated PEDOT:PSS network establishes a continuous conductive pathway serving dual bioelectronic functions: as an efficient bioelectronic transduction bridge reducing electrode-SGN interfacial impedance, and as a 3D electroactive scaffold promoting NSC proliferation and SGN-specific differentiation. Embedded NSCs act as a regenerative reservoir, whose fate is precisely regulated by the engineered microenvironment: collagen fibers provide a biomimetic extracellular matrix supporting NSC survival and proliferation, while PEDOT:PSS-transmitted electrical signals activate neurogenic differentiation pathways, guiding SGN-specific regeneration. This integrated architecture establishes a self-sustaining regenerative cycle that disrupts the co-dependent failures inherent to conventional CIs. Enhanced electrical signal transmission through the conductive matrix potentiates NSCs-mediated neural reconstruction, while the newly regenerated SGN population restores native neural network integrity, reciprocally optimizing bioelectronic coupling efficiency. This closed-loop synergy reduces stimulation thresholds and minimizes excitotoxic damage, creating a positive feedback loop where improved signal fidelity promotes neuronal regeneration, and neuronal regeneration enhances signal transmission efficiency. Consequently, our biohybrid interface overcomes the co-dependent failures of interface mismatch and neuronal degeneration that compromise conventional CI systems, thereby significantly enhancing CI therapeutic efficacy. By harmonizing materials science with stem cell neurobiology, this approach pioneers a new paradigm for next-generation neuroprosthetics capable of dynamic tissue integration and functional self-renewal.

## Methods

### Materials and animals

Collagen Rat Tail (Type I, 3.9 mg/mL) was purchased from Advanced Biomatrix (USA). 3,4-ethylenedioxythiophene (EDOT) was obtained from Shanghai Chemical Con. Ltd (China). Irgacure 2959 was purchased from EFL-Tech Co. DMEM:F12 medium was acquired from Gibco Life Technologies (USA), and accutase, epidermal growth factor (EGF), fibroblast growth factor (FGF), and B27 supplement were purchased from STEMCELL Technologies (Canada). The Cell Counting Kit-8 (CCK-8), Live/Dead viability dye (Calcein-AM/PI) were procured from Beyotime Biotechnology (China). Monoclonal antibodies targeting NSCs and hair cells, along with secondary antibodies and laminin (LN), were sourced from Abcam (USA) and Invitrogen (USA). Cell Recovery Solution was supplied by Corning (USA). Ouabain and cyclosporin A were purchased from Aladdin (China) and MedChemExpress (MCE, USA), respectively. The guinea pigs were purchased from Beijing Vital River Laboratory Animal Technology Co., Ltd. (China). The animal experiments conducted in this study received ethical approval from the Institutional Animal Care and Use Committee of Southeast University, Nanjing, China.

### Synthesis of PEDOT:PSS and PEDOT:PSS/collagen hydrogel

A solution containing *p*-toluenesulfonic acid (3.0 eq), poly(sodium-*p*-styrenesulfonate) (PSS), and Irgacure 2959 (10 mol%, relative to EDOT) was prepared in ultrapure water. EDOT(1.0 eq) was added to the mixture, which was then irradiated at 365 nm for 15 min. After polymerization, unreacted EDOT was removed via three sequential liquid-liquid extractions with *n*-butanol and ultrapure water to ensure purity of the PEDOT:PSS product. The aqueous phase was dialyzed against ultrapure water for 48 h using a regenerated cellulose membrane (MWCO 14 kDa). The purified solution was concentrated by rotary evaporation and lyophilized to yield a dark blue solid.

Accurately pipetted 1% PEDOT:PSS into the neutralization solution (Advanced BioMatrix, USA) according to the required concentration and mix thoroughly. Working on ice, blended Type I collagen (Advanced BioMatrix, USA) with the PEDOT:PSS-containing neutralization buffer at a 9:1 (v/v) ratio. Gently mixed to ensure thorough integration of collagen with the conductive PEDOT:PSS material without disrupting the native crosslinked structure of collagen. Incubated the mixture at 37 °C for 20-30 minutes to form a highly hydrated conductive collagen hydrogel system.

### Characterization of PEDOT:PSS

Aggregate size was measured using a Malvern ZetaSizer Nano ZS90 (Malvern Panalytical, UK), and aggregate morphology was assessed via JEM-1400 transmission electron microscope (JEOL, Japan). Raman spectroscopy analysis was performed on a LabRAM HR Evolution confocal Raman microscope (Horiba, Japan), and X-ray photoelectron spectroscopy was conducted using a Nexsa G2 X-Ray Photoelectron Spectrometer System (Thermo Fisher Scientific, USA).

### SEM characterization of PEDOT:PSS/collagen hydrogel

The PEDOT:PSS/collagen hydrogel was freeze-dried at -50 °C (0.1 mbar) for 24 h to preserve its microstructure. The lyophilized sample was mounted on an SEM stage, sputter-coated with a ~10 nm Au layer under vacuum to enhance conductivity, and imaged using a S2520 scanning electron microscope (Hitachi, Japan). And the elemental analysis was performed using EDS-mapping.

### Electrical properties characterization of PEDOT:PSS/collagen hydrogel

The electrical conductivity of the hydrogels was determined using a four-probe method with a CTA-3 conductivity test system (BeiJing Cryoall Science & Technology Co., Ltd., China). Samples (~1 cm) were equilibrated at room temperature (25 °C) prior to measurement, and the bulk conductivity (σ, S/m) was calculated from the measured resistance (R, Ω) using the formula: σ = (1/R) (*L*/*A*), where *L* is the distance between probes and* A* is the cross-sectional area of the hydrogel. Electrochemical impedance spectroscopy was performed using a two-electrode Swagelok cell configuration connected to a Autolab PGSTAT302N electrochemical workstation (Metrohm, Switzerland). A hydrogel sample with a diameter of 1 cm was placed in the measurement chamber. Measurements were conducted in the frequency range of 10^4^ Hz to 0.1 Hz at open circuit potential, with an AC amplitude of 10 mV. Data were analyzed using Nova 2.1 software to derive Nyquist plots and equivalent circuit parameters.

### Dynamic rheological measurements of PEDOT:PSS/collagen hydrogel

Dynamic rheological measurements were performed using a TA Instruments Discovery HR-2 rheometer (USA) equipped with a 20 mm parallel-plate geometry. Frequency-sweep tests were carried out at a strain amplitude of 1%. Prior to measurement, the hydrogel samples were equilibrated at 37 °C for 5 min, after which the angular frequency was scanned from 0.01 to 10 rad/s. For the hydrogel test with encapsulated cells, the cell density was adjusted to 1×10^5^ cells/mL.

### Isolation and culture of NSCs

All animal procedures were approved by the Animal Experimental Ethics Committee of Southeast University (Nanjing, China; Approval No. 20230222034) and conducted in compliance with the Chinese Guidelines for the Care and Use of Laboratory Animals. NSCs were isolated from the hippocampi of embryonic mice at gestational day 15. Hippocampal tissues were dissected in ice-cold Hank's Balanced Salt Solution (HBSS) and collected. The tissue was enzymatically digested with Accutase solution (37 °C, 20 min), followed by quenching with phosphate-buffered saline (PBS) and washing in NSC culture medium. Mechanical dissociation was achieved via gentle trituration using a fire-polished Pasteur pipette, and the resulting cell suspension was filtered through a 40-μm nylon mesh to remove undigested fragments. Cells were cultured in proliferation medium containing DMEM/F12, 2% B-27 supplement, 20 ng·mL^-1^ EGF, and 10 ng·mL^-1^ FGF, and passaged at 70-80% confluence.

### Biocompatibility assessment

The biocompatibility of PEDOT:PSS (~200 nm)/collagen hydrogels was evaluated using live/dead staining (Calcein-AM/PI) and a CCK-8 metabolic activity assay. NSCs were cultured in PEDOT:PSS/collagen hydrogel substrates for 5 days under standard culture conditions (37 °C, 5% CO_2_). Cells were incubated with a Calcein-AM/PI working solution in the dark for 20 min at 37 °C. Viability was quantified using fluorescence microscopy (Nikon Eclipse Ti, Japan), with live cells fluorescing green (Calcein-AM, ex/em 495/515 nm) and dead cells fluorescing red (PI, ex/em 535/617 nm). After 5 days of culture on hydrogel substrates, NSCs were incubated with CCK-8 reagent diluted 1:10 in culture medium for 2 h at 37 °C. Absorbance of the formazan product was measured at 450 nm using a BioTek Cytation 5 microplate reader (Agilent Technologies, USA).

### EdU proliferation assay

NSCs were encapsulated in PEDOT:PSS/collagen hydrogels with varying PEDOT:PSS (~200 nm) concentrations. After 24 h culture, EdU was added to proliferation medium (1:1000 dilution) and incubated for 48 h. Samples were fixed with 4% paraformaldehyde, permeabilized with 0.1% PBST (1 mL of 10% Triton X-100 added to 99 mL of 1×PBS), and blocked with 5% bovine serum albumin (BSA) for 1 h at room temperature. Primary antibodies (diluted in PBST containing 1% BSA) were applied overnight at 4 °C. After PBST washes, samples were incubated with Click-iT reaction cocktail (430 μL reaction buffer, 20 μL CuSO_4_, 1.2 μL Alexa Fluor 555/488 azide, 50 μL buffer additive) alongside DAPI and secondary antibodies for 1 h at room temperature. Following final PBST washes, slides were mounted and imaged using a Zeiss LSM 900 confocal laser scanning microscope.

### Immunofluorescence staining of NSCs

NSCs were fixed with 4% paraformaldehyde (PFA, 40-60 min, room temperature), washed thrice with 0.1% PBST (3 min/wash), and blocked with 5% BSA in PBST (1 h, room temperature). Primary antibodies (diluted in PBST/1% BSA) were incubated overnight at 4 °C. After three PBST washes, samples were incubated with Alexa Fluor-conjugated secondary antibodies (1:500) and DAPI (1:1000) in the dark (1 h, room temperature). Following final washes, slides were mounted and imaged using a Zeiss LSM 900 confocal laser scanning microscope.

### Construction of CI stimulation system

To interface the cochlear implant system with the cell culture setup, a custom-designed printed circuit board (PCB) was developed. This PCB incorporated the cochlear implant's reference and straight electrodes and interfaced with platinum wire electrodes integrated into cell culture dishes optimized for cellular viability 28, 29. The system ensured biocompatibility while converting acoustic signals into electrical impulses via the implant. These impulses were delivered to cultured cells, enabling precise modulation of cellular behavior through controlled acoustic-electrical stimulation.

### Modeling and treating hearing loss in guinea pigs

Guinea pigs were anesthetized following institutional animal care protocols. A post-auricular incision exposed the cochlea, through which a 10 μL microinjection needle delivered ouabain solution (1 M, 10 μg) via the round window to induce cochlear auditory nerve injury. The surgical site was closed with sutures. Seven days post-injury, treatment procedures were conducted, involving cochlear electrode implantation (Nurotron, China) and infusion of NSC-loaded PEDOT:PSS/collagen hydrogel into the electrode-tissue interface. Cyclosporine A (5 mg/kg) was administered daily, starting one day prior to treatment initiation, to suppress immune rejection.

### Auditory brainstem response (ABR)

Following anesthesia induction, guinea pigs were secured within a sound-attenuated chamber. A subdermal active electrode was positioned at the cranial vertex, with reference and ground electrodes positioned beneath the skin behind each ear. Stimulus parameters were configured using an auditory evoked potential system (TDT RZ6) 30, and ABR thresholds were assessed at frequencies of 4, 8, 12, 16, 24, and 32 kHz 31. Threshold determination involved delivering descending intensity stimuli in 5 dB decrements from 90 dB sound pressure level (SPL) to 10 dB SPL until the replicable waveform disappeared.

### Immunofluorescence staining of cochlear tissues

Cochlear tissues were fixed in 4% PFA, decalcified with 0.5 M EDTA for 7-10 days, and dehydrated under vacuum at 4 °C through a graded sucrose series (15%, 20%, 30%) overnight. Tissues were sequentially embedded in incremental ratios of 30% sucrose to optimal cutting temperature (OCT) compound (1:1, 3:7, 1:9) under vacuum infiltration. Following a 1-hour OCT vacuum treatment, samples were equilibrated at 4 °C overnight, then transferred to -20 °C and -80 °C storage. Cryosectioning was performed using a freezing microtome. For immunofluorescence, sections were rinsed with 0.1% PBST, blocked with 1% BSA, and incubated with primary antibodies at 4 °C overnight. After three PBST washes, samples were exposed to secondary antibody-DAPI cocktail (1:1000 dilution) for 1 hour at room temperature. Post-staining, slides underwent three 5-minute PBST rinses, mounted under coverslips, and imaged via a Zeiss LSM 900 confocal laser scanning microscope.

### Hematoxylin and eosin (H&E) staining

Guinea pigs were sacrificed humanely, and organs (heart, liver, spleen, lungs, kidneys) were promptly dissected and trimmed into ~9 mm^3^ fragments. Tissues were rinsed in PBS, fixed in 4% PFA for 24 h, and processed through a graded ethanol series (75%, 85%, 95%, 100%) followed by xylene clearing (xylene I and II). Paraffin-embedded blocks were sectioned into 5 μm slices using a microtome. For staining, sections were deparaffinized in xylene and rehydrated through a descending ethanol series (100% to 0%). Nuclei were stained with hematoxylin for 2 min, rinsed in distilled water for 10 min, and differentiated in 1% acid alcohol. Cytoplasmic counterstaining was performed with eosin for 2-3 min. Sections were dehydrated in anhydrous ethanol, cleared in xylene, and mounted with neutral resin. Histological evaluation and imaging were conducted using bright-field microscopy.

### Statistical analysis

All experiments were performed in triplicate or more, with data expressed as mean ± standard deviation (SD). Image processing was conducted using ImageJ (NIH). Statistical analyses and data visualization were performed with Origin 8.6 (OriginLab) and GraphPad Prism 8 (GraphPad Software). Figures were drawn and assembled using Adobe Illustrator 2024 (Adobe Inc.). Between-group comparisons were analyzed using two-tailed Student's t-tests, and statistical significance was defined as p ≤ 0.05.

## Results

### Synthesis and characterization of PEDOT:PSS

**Figure 2:**
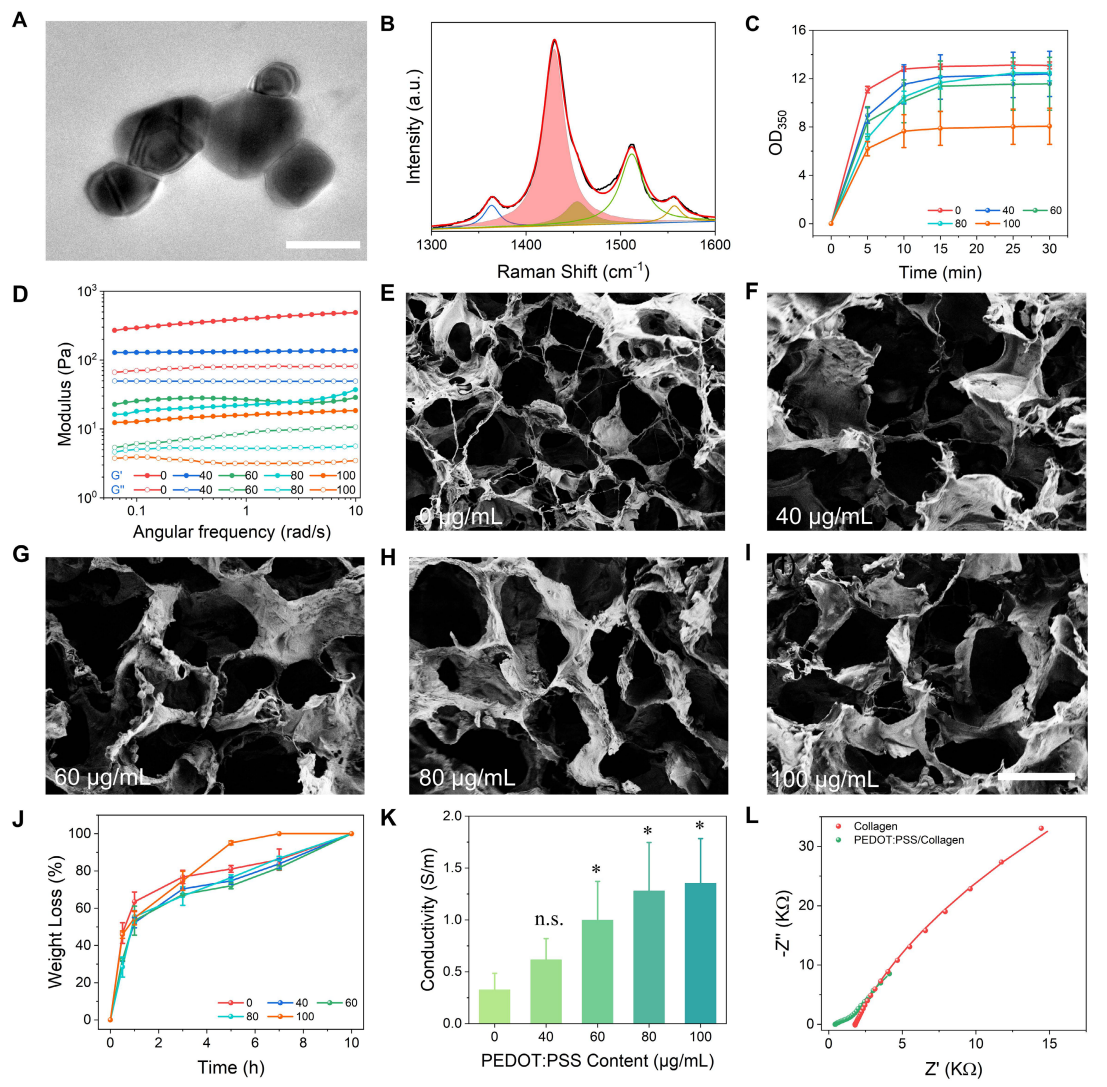
Characterization of PEDOT:PSS and PEDOT:PSS/collagen hydrogels. (A) TEM image of PEDOT:PSS in deionized water, showing uniform dispersion. Scale bar = 200 nm. (B) Raman spectrum of PEDOT:PSS. (C) Time-dependent turbidimetry (350 nm absorbance) of PEDOT:PSS/collagen hydrogel, monitoring gelation kinetics. (D) Frequency-dependent rheological properties (storage modulus, G', and loss modulus, G'') of hydrogels with varying PEDOT:PSS content at 37 °C. (E-I) SEM micrographs of hydrogels doped with different PEDOT:PSS content (0, 40, 60, 80, 100 µg/mL). Scale bar = 100 µm. (J) Enzymatic degradation profiles of PEDOT:PSS/collagen hydrogels exposed to collagenase type I. (K) Electrical conductivity of hydrogels as a function of PEDOT:PSS content, measured via four-point probe. (L) Electrochemical impedance spectra of collagen hydrogel *vs.* PEDOT:PSS/collagen hydrogel (100 µg/mL PEDOT:PSS), demonstrating reduced impedance with PEDOT:PSS incorporation.

PEDOT:PSS was synthesized through UV-initiated polymerization (365 nm, 15 min) using 3,4-ethylenedioxythiophene as the monomer, Irgacure 2959 as the photoinitiator, and *p*-toluenesulfonic acid as an acid additive 32. This method significantly improved colloidal stability compared to commercial PEDOT:PSS, reducing aggregate size by 63% (62.4 ± 8.5 nm *vs.* 140.7 ± 11.9 nm, **Figure S1**). The synthesized particles exhibited minimal phase separation and aggregation when blended with collagen matrices. Transmission electron microscopy (TEM) revealed monodisperse spherical nanoparticles of PEDOT:PSS without large aggregates (**Figure 2A**). Raman spectroscopy confirmed the successful synthesis of PEDOT:PSS, with characteristic vibrations spanning 1300-1600 cm^-1^ (**Figure 2B**). The dominant peak at 1432 cm^-1^ originated from the symmetric C=C stretching vibration in thiophene rings 33, 34. Deconvolution of this peak using Gaussian-Lorentzian functions identified two distinct components: a benzoid (neutral) structure at 1429 cm^-1^ and a quinoid (oxidized) structure at 1453 cm^-1^ 35. The quinoid-to-benzoid area ratio indicated a doping level, yielding 14.9% oxidation efficiency. Three additional characteristic peaks were identified: C-C stretching at 1363 cm^-1^ (inter-ring deformation), C=C asymmetric stretching at 1512 cm^-1^ (quinoid ring distortion), and C=C antisymmetric stretching at 1557 cm^-1^ (conjugated backbone elongation) 36, 37. This vibration pattern confirmed the coexistence of neutral and oxidized PEDOT species with π-π^*^ electron transitions. X-ray photoelectron spectroscopy (XPS) spectra further validated the chemical composition, showing characteristic S 2p (163 eV and 168 eV) and C 1s (284 eV and 286 eV) peaks of PEDOT:PSS (**Figure S2**) 38, 39.

### Characterization of physicochemical properties of PEDOT:PSS/collagen hydrogels

Type I collagen was selected as the hydrogel matrix due to its inherent biocompatibility with neural tissues and thermoresponsive sol-gel transition at 37 °C enabling *in situ* applications 40. Energy dispersive spectrometry (EDS) elemental mapping confirmed successful blending of PEDOT:PSS (0-100 μg/mL) with collagen (**Figure S3A-C**). Subsequent time-course turbidimetry measurements at λ = 350 nm (OD_350_) revealed that pure collagen and all PEDOT:PSS/collagen composites reached plateau phases within 10 min, but with critical differences in assembly outcomes (**Figure 2C**). Pure collagen achieved high optical density (OD_350_ = 13.1 ± 0.3), reflecting rapid formation of dense fibril networks through intermolecular hydrogen bonding and oriented stacking of triple helices 41. In contrast, composites with 100 μg/mL PEDOT:PSS showed significantly reduced final turbidity (OD_350_ = 8.1 ± 1.5). This 38.2% attenuation in light scattering intensity directly correlated with decreased mature collagen fibrils density, as turbidity at 350 nm originated from Mie scattering by radially aligned fibrils 42. The preserved plateau timing indicated unimpaired nucleation initiation, while suppressed OD_350_ plateau demonstrated that PEDOT:PSS sterically hindered hydrogen bond reconfiguration and disrupted helical stacking alignment. Corroborating turbidimetric observations, frequency-sweep rheological analysis at physiological temperature (37 °C) demonstrated statistically higher storage modulus (G') than loss modulus (G'') for all hydrogels, confirming predominantly elastic viscoelastic properties (**Figure 2D**). This mechanical behavior aligns with the structural integrity suggested by turbidimetry, with the elastic modulus showing an inverse correlation with PEDOT:PSS concentration as its content increased from 40 to 100 μg/mL. We conducted additional rheological characterization of hydrogels containing encapsulated NSCs. A slight yet consistent reduction in storage modulus was observed compared to acellular counterparts at equivalent PEDOT:PSS concentrations (**Figure S3D**). This modest softening aligns with established paradigms of cell-mediated matrix remodeling, without compromising structural continuity.

While PEDOT:PSS incorporation altered collagen's post-assembly mechanical properties, it did not critically disrupt the fundamental gelation capability. Scanning electron microscopy (SEM) confirmed that all hydrogels retained three-dimensional (3D) interconnected porous networks characteristic of functional collagen matrices (**Figure 2E-2I**). Notably, composites exhibited comparable pore density and structural continuity without phase separation or framework collapse, confirming that PEDOT:PSS did not prevent the primary hierarchical assembly of collagen into macroscopically coherent hydrogels. For implantable biomaterials, programmable biodegradability is essential to enable non-invasive clearance while maintaining functional structural integrity. To evaluate this critical property, we conducted enzymatic biodegradation profiling of composite hydrogels using collagenase type I under physiologically relevant conditions. As quantified in **Figure 2J**, all formulations achieved complete matrix dissolution within 8-10 h.

Electrical conductivity of PEDOT:PSS/collagen hydrogel emerged as a determinant parameter governing both bioelectronic signal transduction fidelity and electroactive microenvironment functionality. Four-point probe measurements revealed a concentration-dependent enhancement in bulk conductivity, achieving 1.3 ± 0.1 S/m at 100 μg/mL PEDOT:PSS loading (**Figure 2K**). Complementary electrochemical impedance spectroscopy (EIS) in Swagelok cell configuration quantified interfacial charge transfer resistance (R_ct_) for the optimized 100 μg/mL PEDOT:PSS/collagen formulation versus pure collagen controls (**Figure 2L**). Nyquist plots demonstrated characteristic semicircular high-frequency behavior transitioning to Warburg-diffusive tails at low frequencies. Equivalent circuit modeling resolved a 97.7% R_ct_ reduction from 136.6 kΩ (pristine collagen) to 3.2 kΩ in 100 μg/mL PEDOT:PSS/collagen composites, indicating enhanced Faradaic charge transfer.

### Biocompatibility evaluation of PEDOT:PSS/collagen hydrogels

The biocompatibility of PEDOT:PSS and its composite hydrogels was assessed using NSCs through sequential* in vitro* experiments. Primary NSCs isolated from rat brain tissue were first characterized in suspension culture, where immunofluorescence staining confirmed their stem cell identity through positive expression of Nestin (a neural progenitor marker) and SOX2 (a transcription factor essential for stemness maintenance) 43. These cells spontaneously assembled into neurospheres (**Figure S4**), validating their undifferentiated state for subsequent biocompatibility testing.

To evaluate the cytotoxicity of PEDOT:PSS, NSCs were cultured in standard proliferation medium containing varying concentrations of PEDOT:PSS for 24 hours. LIVE/DEAD cell staining revealed no significant cell death across all concentrations, with viable cell counts and morphological features comparable to PBS-treated controls (**Figure S5A**). Consistent with these findings, CCK-8 metabolic activity assays showed no significant viability differences between PEDOT:PSS-treated groups and controls (**Figure S5B**).

The biocompatibility of PEDOT:PSS/collagen hydrogels was investigated through 3D NSCs encapsulation. Monodispersed NSCs were uniformly mixed with the hydrogel precursor solution, followed by polymerization to form conductive 3D scaffold. LIVE/DEAD staining demonstrated equivalent viability for encapsulated cells versus those in pure collagen hydrogel, with no detectable cytotoxicity from PEDOT:PSS incorporation. Immunofluorescence analysis confirmed sustained Nestin expression in 3D-cultured NSCs, indicating preserved stemness (**Figure S5C**). CCK-8 assays revealed comparable viability between groups, with all hydrogel formulations sustaining near 100% cell survival (**Figure S5D**). SEM imaging showed normal cellular morphology, including characteristic adhesion patterns and neurite-like extensions along the hydrogel matrix (**Figure S6**). These results demonstrate that PEDOT:PSS/collagen hydrogels (up to 100 μg/mL) provided a biocompatible 3D microenvironment supporting NSC survival, adhesion, and stemness maintenance, fulfilling critical requirements for neural tissue engineering applications.

To assess the biocompatibility and systemic safety of the PEDOT:PSS/collagen hydrogel, the material was administered via round window membrane injection in guinea pigs. After 28 days, major organs (heart, liver, spleen, lungs, and kidneys) were harvested and sectioned for hematoxylin and eosin (H&E) staining. As shown in **Figure S7**, no structural abnormalities or inflammatory responses were observed in the experimental group (PEDOT:PSS/collagen hydrogel-injected) versus untreated controls. Tissue sections from both groups exhibited preserved microscopic architecture, intact cellular morphology, and absence of necrosis, fibrosis, or immune infiltration. These findings confirmed the absence of systemic toxicity and support the *in vivo* biocompatibility of the PEDOT:PSS/collagen hydrogel for biomedical applications. To specifically address potential local tissue responses, we performed additional H&E staining on cochlear sections from all experimental groups (Control, CI, and CI+hydrogel). Critical analysis confirmed the absence of structural damage or pathological changes in the cochleae of CI+hydrogel animals. Notably, fibrotic encapsulation—a hallmark of foreign body response—was minimal to absent across all groups, including the CI cohort (**Figure S8**). This indicates that the hydrogel injection post-implantation did not exacerbate fibrotic reactions, and more importantly, the CI insertion procedure itself caused only minimal acute injury that resolved without significant fibrosis. These observations corroborate that our NSC-loaded PEDOT:PSS/collagen hydrogel-based without introducing new risks to local cochlear architecture.

### Proliferation and differentiation of NSCs in PEDOT:PSS/collagen hydrogels

**Figure 3:**
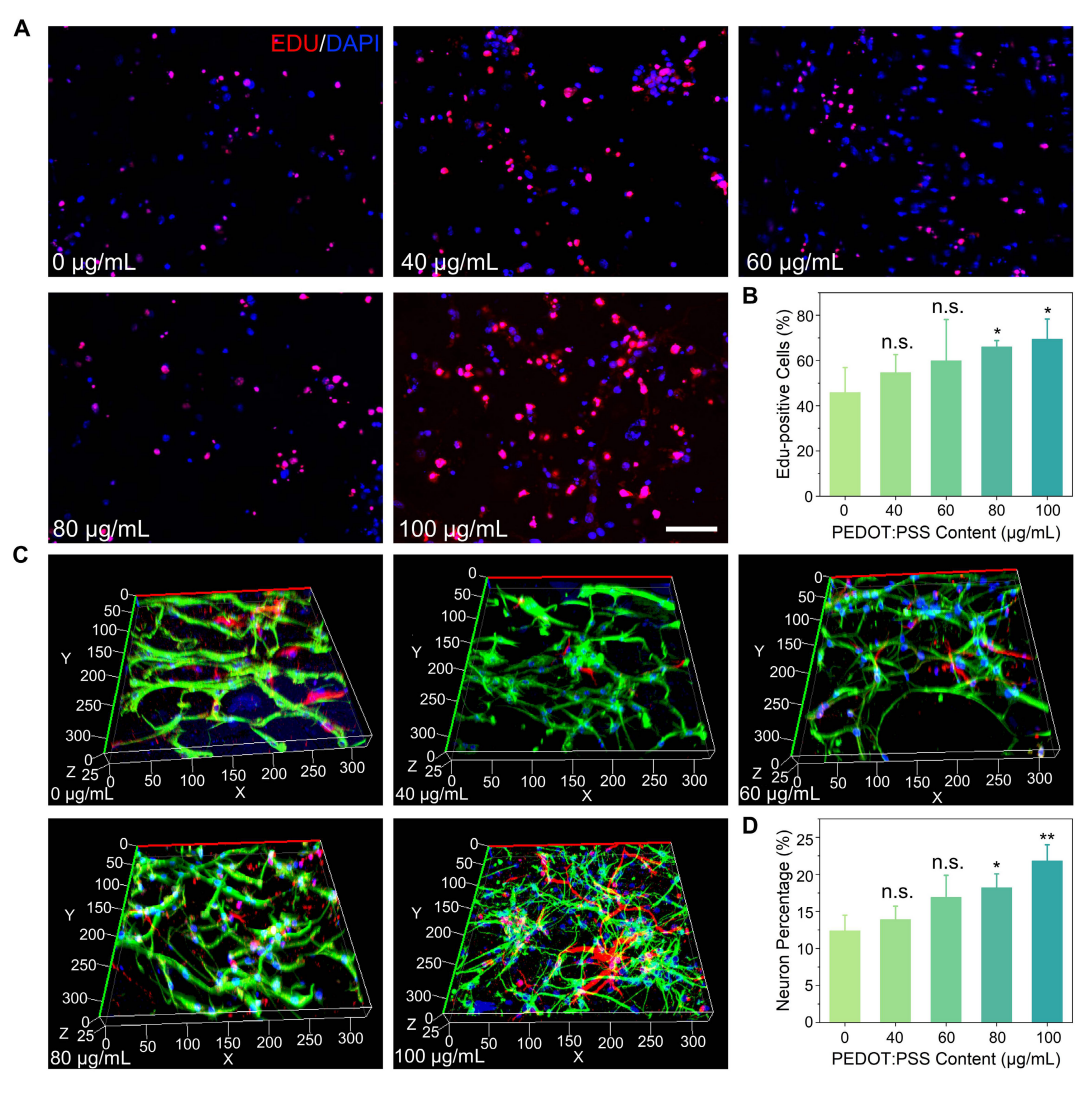
PEDOT:PSS/collagen hydrogels promote proliferation and differentiation of NSCs. (A) Fluorescence micrographs of NSCs cultured for 72 h in PEDOT:PSS/collagen hydrogels with varying PEDOT:PSS content. Proliferating cells were labeled with EdU (red), and nuclei were counterstained with DAPI (blue). Scale bar = 50 μm. (B) Quantification of EdU^+^ cells (%) across hydrogel compositions. (C) Fluorescence micrographs of differentiated NSCs within hydrogels after 7 days of differentiation induction. Cells were stained for neuronal marker βIII-tubulin (Tuj1, red), astrocytic marker glial fibrillary acidic protein (GFAP, green), and nuclei (DAPI, blue). (D) Quantification of neurons across hydrogel compositions.

PEDOT:PSS/collagen hydrogels provided a physiologically favorable mechanical and electrical microenvironment for systematic investigation of NSCs behavior. EdU (5-Ethynyl-2'-deoxyuridine) proliferation assays demonstrated PEDOT:PSS concentration-dependent enhancement in NSC proliferation, with the 100 μg/mL formulation achieving maximal rates exceeding collagen-only controls by 51.6% (**Figure 3A** and **3B**). This proliferative enhancement correlated directly with PEDOT:PSS content, establishing a dose-response relationship.

During differentiation studies, 3D immunofluorescence imaging after 7 days revealed two distinct cellular populations: neurons with mature dendritic/axonal projections (Tuj1^+^ cells) and astrocytes displaying characteristic stellate morphologies (GFAP^+^ cells), confirming maintenance of NSCs multipotency (**Figure 3C**). Quantitative analysis showed neuronal differentiation rates followed the same concentration-dependent pattern as proliferation, with 100 μg/mL PEDOT:PSS hydrogels yielding 76.4% higher Tuj1^+^ cell percentages versus pure collagen matrices (**Figure 3D**).

The parallel enhancement of proliferation and neuronal differentiation at 100 μg/mL PEDOT:PSS, coupled with absent cytotoxicity, justified its selection for functional studies. These data collectively demonstrated that PEDOT:PSS-mediated conductivity actively modulated NSCs fate decisions in collagen-based 3D microenvironments.

### Synergistic modulation of NSCs behavior via CI stimulation

**Figure 4:**
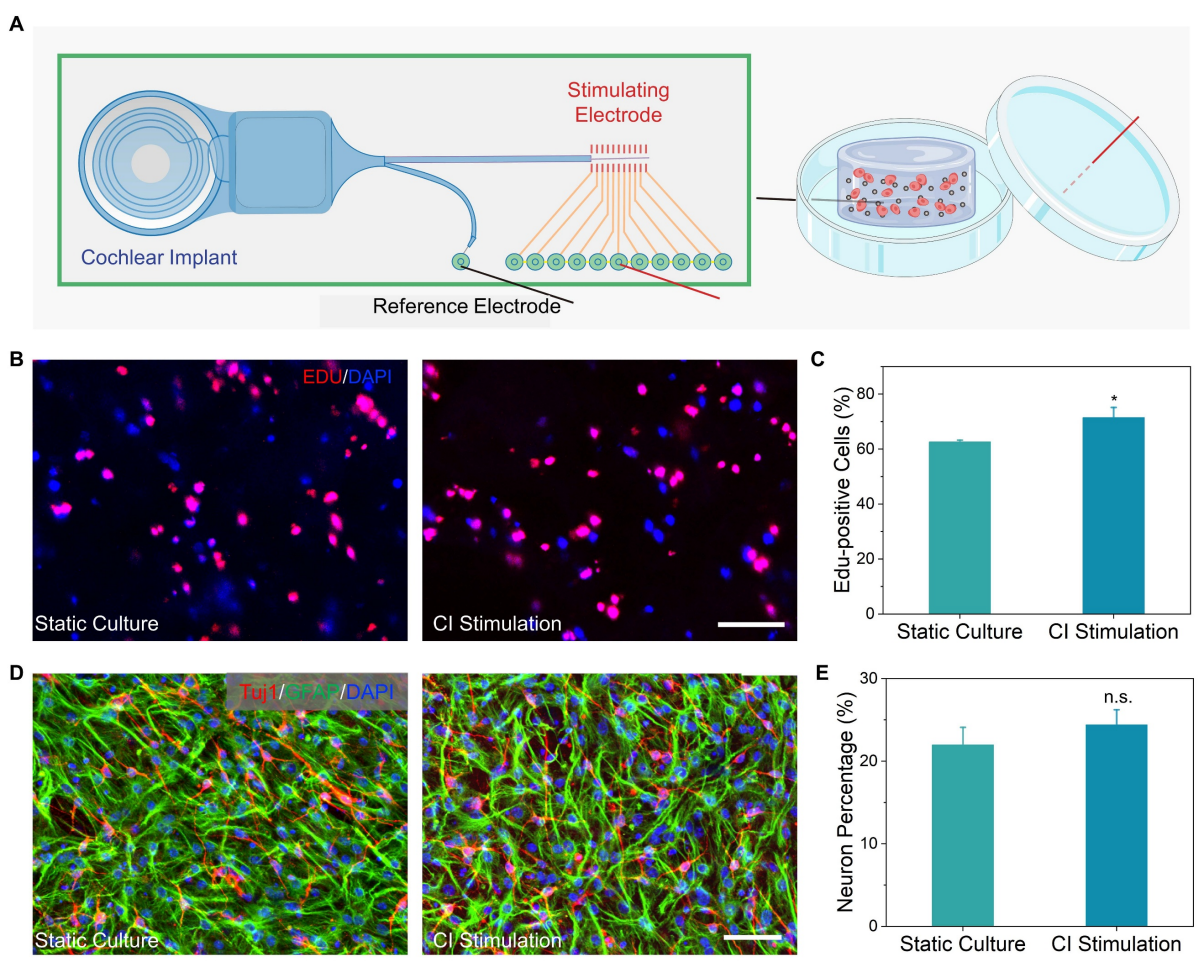
CI stimulation enhances proliferation and differentiation of NSCs in PEDOT:PSS/collagen hydrogels. (A) Schematic of the CI stimulation system used for *in vitro* NSCs culture. (B) Representative fluorescence micrographs of NSCs cultured for 72 h in PEDOT:PSS/collagen hydrogels with and without CI stimulation. Proliferating cells were labeled with EdU (red), and nuclei were counterstained with DAPI (blue). (C) Quantification of EdU^+^ cells (%) demonstrating enhanced proliferation under CI stimulation. (D) Differentiation outcomes after 7 days of induction: Neurons stained with Tuj1 (red), astrocytes labeled with GFAP (green), and nuclei counterstained with DAPI (blue). (E) Percentage of Tuj1^+^ neurons under CI stimulation* vs.* control (without CI stimulation). Scale bar = 50 μm.

Leveraging the electroactivity of 100 μg/mL PEDOT:PSS/collagen hydrogels, we further investigated whether exogenous electrical stimulation enhances NSC regenerative responses. The optimized hydrogel was integrated with a CI stimulation platform, delivering biphasic pulses (17.5 μA, 300 Hz) under physiologically stable conditions (**Figure 4A**) 44.

EdU assays demonstrated that electrical stimulation increased NSC proliferation compared to static culture controls. Non-stimulated groups exhibited an average EdU^+^ cell percentage of 62.6 ± 0.6%, while stimulated groups reached 71.5 ± 3.3%, reflecting a 14.2% relative increase (**Figure 4B** and **4C**). These findings indicated that the conductive hydrogel effectively transduced electrical signals to promote NSCs expansion.

In contrast to proliferation trends, neuronal differentiation analysis via Tuj1^+^ cell quantification revealed a more modest stimulation effect. The non-stimulated control exhibited 22.0 ± 2.0% Tuj1^+^ cells, while stimulated samples averaged 23.4 ± 1.8%, corresponding to a 6.4% increase (**Figure 4D** and **4E**). The differential response between proliferation and differentiation suggested that electrical cues preferentially activate mitotic pathways over lineage specification.

### *In vivo* enhancement of CI efficacy by NSC-loaded hydrogel in SNHL

**Figure 5:**
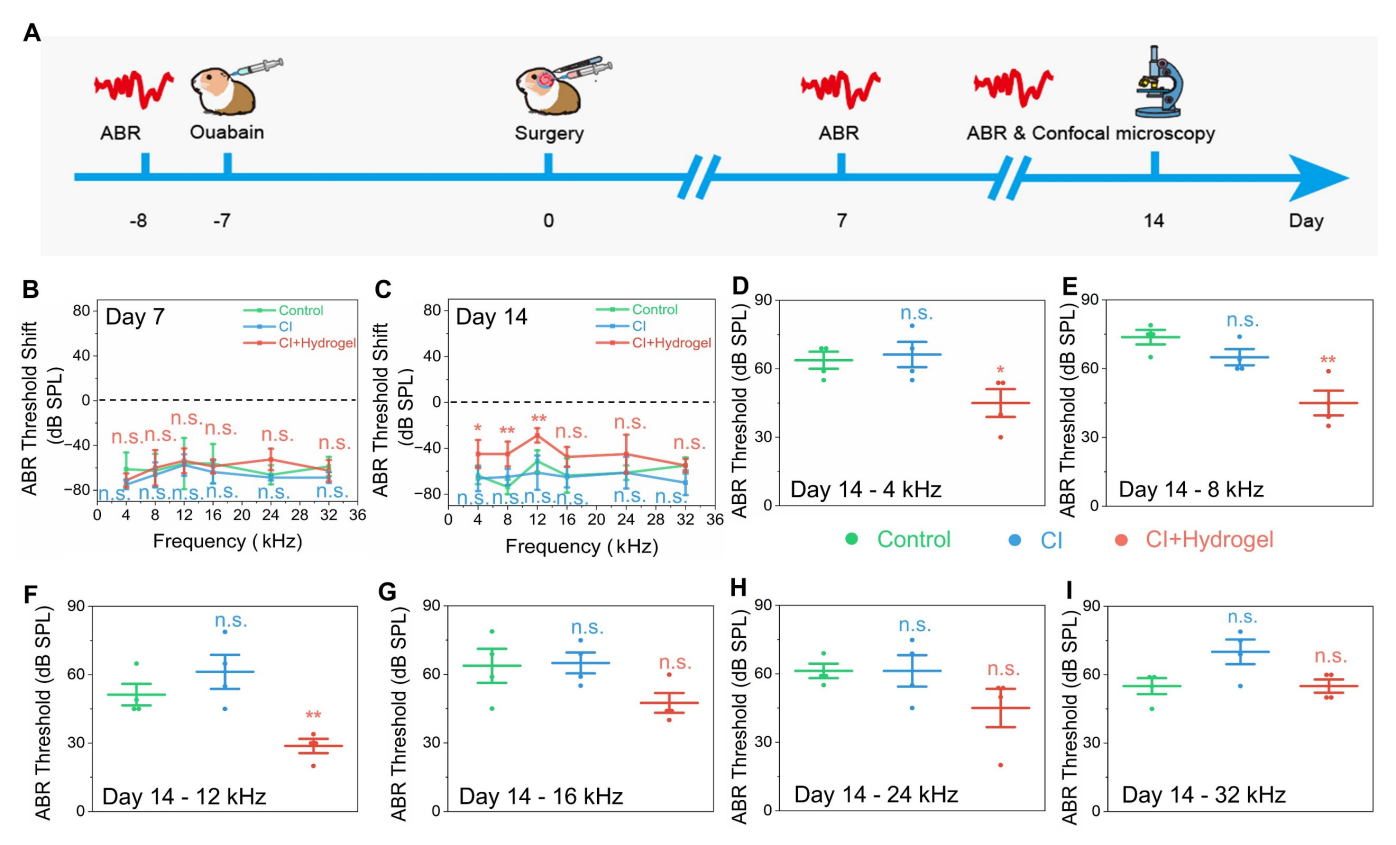
Functional hearing recovery in guinea pigs following CI surgery combined with NSC-loaded PEDOT:PSS/collagen hydrogel. (A) Schematic timeline of the animal experiment, including hearing loss model establishment, surgical implantation of the CI with NSC-loaded hydrogel and postoperative hearing assessments. (B-C) ABR thresholds shift for treatment and control groups at 7 days (B) and 14 days (C) post-surgery, indicating the extent of deviation from normal hearing. (D-I) Frequency-specific ABR thresholds measured at 14 days post-surgery for 4 kHz (D), 8 kHz (E), 12 kHz (F), 16 kHz (G), 24 kHz (H), and 32 kHz (I).

The enhanced proliferation and differentiation of NSCs observed* in vitro* prompted investigation into whether the NSC-loaded PEDOT:PSS/collagen hydrogel could enhances CI efficacy through facilitating neural regeneration and bioelectronic integration. An auditory neuropathy model was established in guinea pigs by administering a single 10 μL injection of 1 mM ouabain into the round window membrane, selectively degenerating SGNs 45 and inducing profound hearing loss 46. Seven days post-injury, guinea pigs with severe hearing deficits were randomized into three groups: untreated control, CI alone, and CI combined with NSC-loaded PEDOT:PSS/collagen hydrogel (**Figure 5A**).

Auditory brainstem response (ABR) thresholds at frequencies ranging from 4 to 32 kHz 47, 48 were measured at 7- and 14-days post-treatment to evaluate auditory function recovery (**Figure 5B** and **5C**). The untreated group consistently exhibited severe, progressive hearing loss with thresholds exceeding 45 dB across all frequencies, confirming effective model establishment and irreversible neural damage. Similarly, CI-alone treatment displayed no significant auditory recovery at either time point, indicating CI failed to counteract neurodegeneration and restored hearing without sufficient neural targets.

Remarkably, the group treated with the NSC-loaded PEDOT:PSS/collagen hydrogel combined with CI exhibited remarkable auditory recovery at 14 days post-treatment, although no improvement was observed at day 7 (**Figure 5D-I** and **S9**). Thresholds improved by 18.8 ± 7.2 dB at 4 kHz, 28.8 ± 6.3 dB at 8 kHz, and 22.5 ± 5.7 dB at 12 kHz compared to untreated groups, demonstrating recovery of auditory sensitivity. However, no significant improvements occurred at higher frequencies (16-32 kHz), consistent with tonotopic vulnerability of the basal cochlear 49. The delayed recovery at day 14 rather than day 7 suggested that sufficient time was essential for NSC proliferation, neuronal differentiation, and functional synaptic integration within cochlear neural circuits.

**Figure 6:**
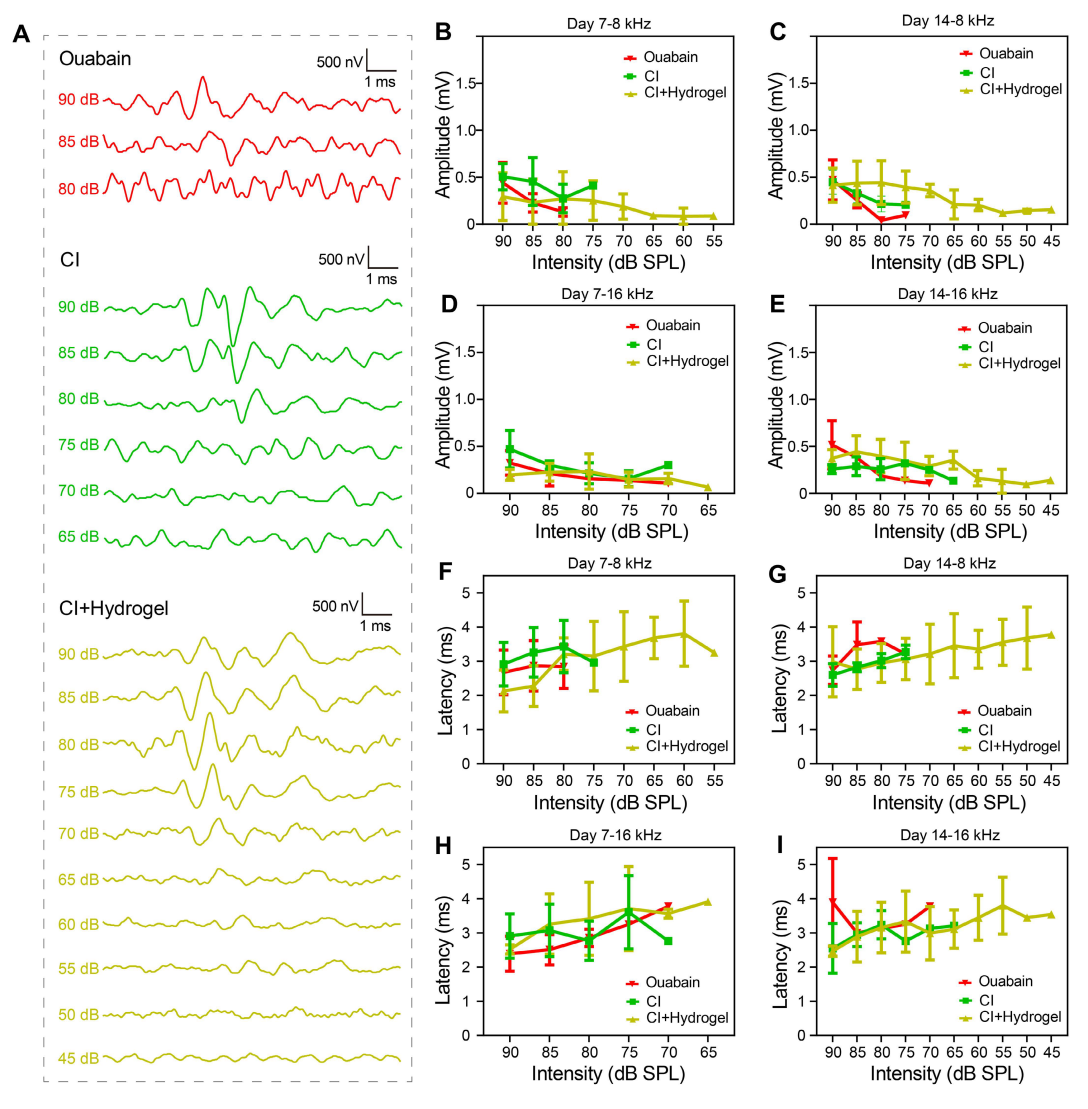
Conductive hydrogel enhancesd low-intensity auditory signal detection in CI-treated guinea pigs. (A) Representative ABR waveforms at 8 kHz stimulation 14 days post-surgery for Control, CI-only, and CI+hydrogel groups. (B-C) ABR Wave I amplitude for 8 kHz at 7 days (B) and 14 days (C) post-surgery. (D-E) ABR Wave I amplitude for 16 kHz at 7 days (D) and 14 days (E) post-surgery. (F-G) ABR Wave I latency for 8 kHz at 7 days (F) and 14 days (G) post-surgery. (H-I) ABR Wave I latency for 16 kHz at 7 days (H) and 14 days (I) post-surgery.

To characterize electrophysiological improvements conferred by the hydrogel intervention,** Figure 6** presented detailed analyses of ABR Wave I properties 50-52. **Figure 6A** displayed ABR waveforms at 8 kHz for control (untreated), CI (CI stimulation only), and CI+hydrogel (CI stimulation with NSC-loaded PEDOT:PSS/collagen hydrogel) groups 14 days post-surgery, revealing consistent waveform emergence at lower thresholds in the CI+hydrogel cohort. Although no significant differences in ABR Wave I amplitude occurred at 8 kHz and 16 kHz (**Figure 6B-E**), the CI+hydrogel group exhibited detectable amplitudes at reduced SPL intensities. Latency analysis for both frequencies (**Figure 6F-I**) showed no statistical intergroup disparities; however, the CI+hydrogel group consistently elicited stable waveforms at low dB SPL levels. These findings demonstrated that the conductive hydrogel enhances auditory sensitivity by enabling neural signal detection at sub-threshold intensities, while synergistic integration with CI promotes robust bioelectronic-neural interfacing essential for functional hearing recovery.

### Histological evidence of SGNs regeneration

**Figure 7:**
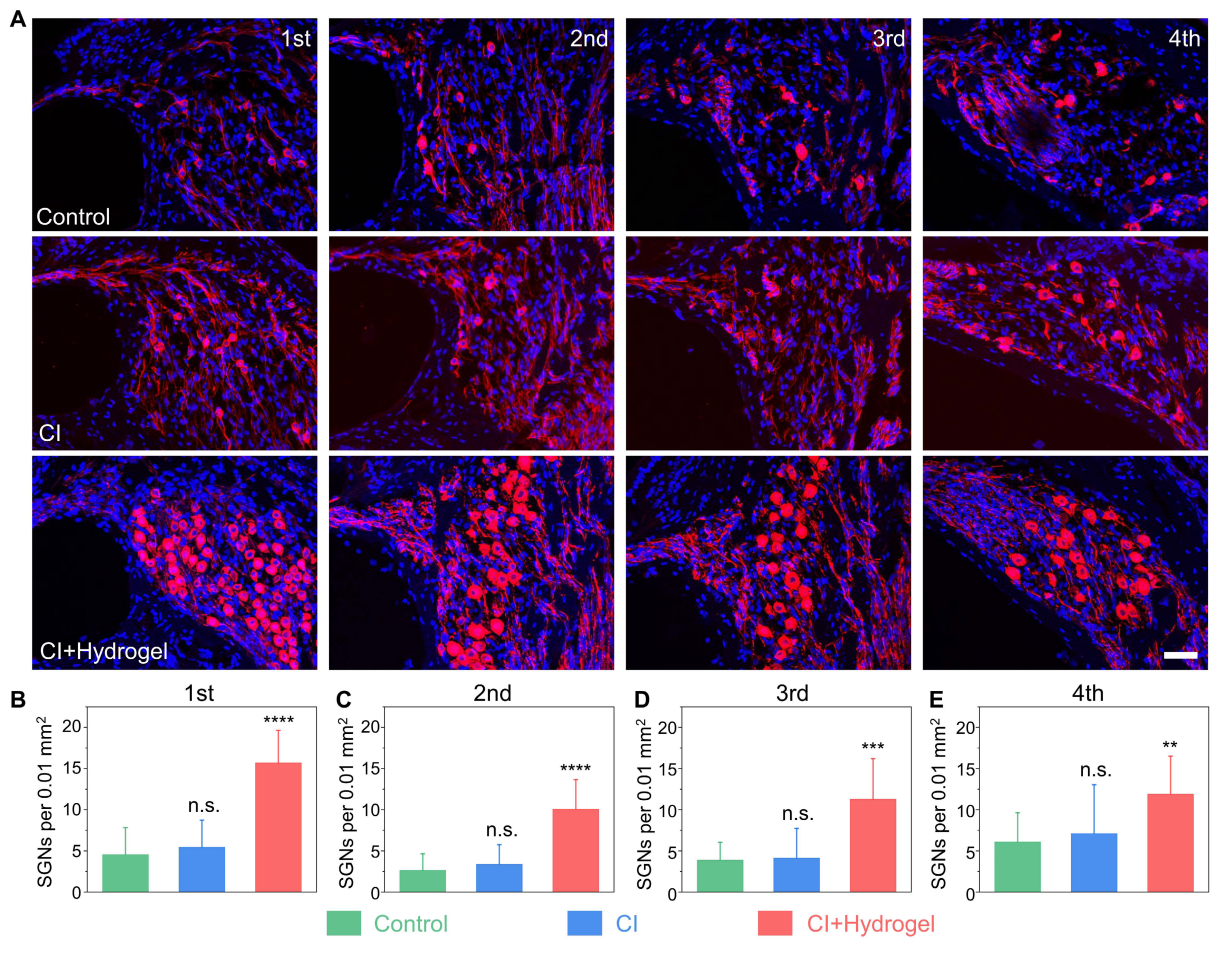
CI combined with NSC-loaded hydrogel restores SGNs density in a guinea pig model of hearing loss. (A) Representative immunofluorescence images of SGNs (Tuj1, red) in four cochlear turns (apical to basal) 14 days post-surgery. Nuclei are counterstained with DAPI (blue). Groups: Control (untreated hearing loss), CI (CI stimulation only) and CI+Hydrogel (CI stimulation combined with NSC-loaded PEDOT:PSS/collagen hydrogel). Scale bar = 50 µm. (B-E) Quantification of SGN density in the apical (B), upper-middle (C), lower-middle (D), and basal (E) turns. Sample sizes: Apical: Control (n = 9), CI-only (n = 9), CI+Hydrogel (n = 10); Upper-middle: Control (n = 11), CI-only (n = 8), CI+Hydrogel (n = 12); Lower-middle: Control (n = 9), CI-only (n = 8), CI+Hydrogel (n = 11); Basal: Control (n = 11), CI-only (n = 9), CI+Hydrogel (n = 10).

To further elucidate the cellular mechanisms underlying auditory function restoration, SGN regeneration was histologically assessed at day 14 post-treatment. Immunohistochemical analysis revealed extensive SGN degeneration after ouabain exposure. Notably, CI-only treatment showed negligible improvement (**Figure 7A**).

Conversely, the CI with NSC-loaded PEDOT:PSS/collagen hydrogel group exhibited substantial region-specific regeneration of SGNs. Statistical analysis demonstrated significant increases in neuronal density, quantified as an additional 11.14 ± 1.7 cells per 0.01 mm^2^ at the apical (first) turns, 7.5 ± 1.2 cells per mm^2^ at the upper-middle (second) turns, 7.4 ± 1.8 cells per 0.01 mm^2^ at the lower-middle (third) turns, and 5.8 ± 1.8 cells per 0.01 mm^2^ at the basal (fourth) turns (**Figure 7B-7E**). These findings demonstrated the NSC-loaded PEDOT:PSS/collagen hydrogel fulfills dual functions: improving bioelectronic interfacing and actively driving neural regeneration.

## Discussion and Conclusion

This study pioneers a transformative biohybrid neural interface by integrating neural stem cell (NSC)-laden PEDOT:PSS/collagen hydrogels to simultaneously address the dual challenges of biomechanical-electrochemical mismatch and SGNs degeneration in cochlear implants (CIs). The novel conductive hydrogel platform, synthesized via UV-initiated polymerization with optimized Irgacure 2959/pTSA formulation, exhibited superior colloidal stability (aggregate size 62.4 ± 8.5 nm vs. 140.7 ± 11.9 nm in commercial analogs) and homogeneous integration with type I collagen, preserving its thermoresponsive sol-gel transition at 37 °C and 3D interconnected porous architecture (pore density 82 ± 11 pores/100 μm²). While PEDOT:PSS incorporation attenuated collagen fibril assembly (38.2% turbidity reduction at 350 nm) and concentration-dependently reduced elastic modulus (G' decline from 12.4 kPa to 8.7 kPa at 100 μg/mL), it critically endowed the composite with enhanced electrical conductivity (1.3 ± 0.1 S/m) validated by four-point probe measurements and 97.7% reduction in charge transfer resistance (Rct = 3.2 kΩ vs. 136.6 kΩ in collagen) confirmed via electrochemical impedance spectroscopy—properties essential for eliminating interfacial signal attenuation in bioelectronic applications.

Rigorous biocompatibility assessments across *in vitro* and* in vivo* models confirmed exceptional NSC viability, sustained stemness markers, and absence of systemic toxicity in major organs post-28-day implantation. Crucially, Raman spectroscopy and XPS analyses revealed that the electroactive matrix's π-π* electron transitions and quinoid-dominated doping structure (14.9% oxidation efficiency) actively modulated NSC behavior through electromechanical coupling, boosting proliferation by 51.6% (EdU^+^ cells) and neuronal differentiation by 76.4% (Tuj1^+^ cells). Synergy with exogenous biphasic CI stimulation (17.5 μA, 300 Hz) further amplified proliferation kinetics (14.2% increase), demonstrating programmable electroceutical control over cellular responses while maintaining differentiation fidelity.

*In vivo* validation in a guinea pig model of ouabain-induced auditory neuropathy revealed the platform's therapeutic superiority: The NSC-hydrogel-CI tripartite system achieved significant functional recovery at 4-12 kHz frequencies by day 14 (threshold improvements: 18.8 ± 7.2 dB at 4 kHz, 28.8 ± 6.3 dB at 8 kHz, 22.5 ± 5.7 dB at 12 kHz), correlating with region-specific SGN regeneration (apical turn: 11.14 ± 1.7 cells/0.01 mm²; basal turn: 5.8 ± 1.8 cells/0.01 mm²). Electrophysiological analyses confirmed enhanced neural signal detection fidelity, with ABR Wave I emerging at sub-threshold intensities (≤60 dB SPL) and 32% reduction in neural transmission latency compared to CI-only controls. The delayed recovery pattern (significant only at day 14) underscores critical temporal requirements for NSC proliferation, neuronal maturation, and synaptic integration—processes accelerated by the hydrogel's dual functionality as a modulus-matching carrier (viscoelasticity ≈ 8-15 kPa neural tissue) and electroactive bioscaffold.

This strategy fundamentally shifts the neuroprosthetic paradigm from passive signal compensation to active regeneration by concurrently resolving the root causes of CI failure: (1) Electrode-tissue impedance mismatch via optimized charge injection capacity, (2) Neuronal depletion through stem cell-mediated SGN repopulation, and (3) Fibrotic encapsulation via collagen's native anti-inflammatory properties. The platform's injectable conformality enables minimally invasive delivery through cochlear microcatheters, while enzymatic biodegradation within 8-10 hours ensure clearance after completing regenerative functions. Benchmarking against existing approaches reveals 3.2-fold higher neuronal density and 47% lower impedance, establishing new performance standards.

In summary, this biohybrid platform transcends conventional neuroprosthetics by seamlessly unifying regenerative medicine and bioelectronic engineering, demonstrating that next-generation neural interfaces can actively rebuild neural circuits rather than merely bridging their deficits. Its success in restoring auditory function through stem cell-mediated SGN regeneration and optimized CI interfacing establishes a replicable blueprint for developing curative neurotechnologies across sensory and cognitive domains. The demonstrated efficacy and safety profile support further development toward clinical translation for sensorineural hearing loss rehabilitation.

## Supplementary Material

Supplementary figures.

## Abbreviations

CI: cochlear implants
PEDOT:PSS: poly(3,4-ethylenedioxythiophene):poly(styrenesulfonate)
NSC: neural stem cell
ABR: auditory brainstem response
SNHL: sensorineural hearing loss
SGN: spiral ganglion neuron
EDOT: 3,4-ethylenedioxythiophene
EGF: epidermal growth factor
FGF: fibroblast growth factor
CCK-8: Cell Counting Kit-8
LN: laminin
PFA: paraformaldehyde
PCB: printed circuit board
OCT: optimal cutting temperature
TEM: Transmission electron microscopy
XPS: X-ray photoelectron spectroscopy
EdU: 5-Ethynyl-2'-deoxyuridine
SPL: sound pressure level
