# Supplementary material for: Neural stem cell-loaded biohybrid hydrogel improves cochlear implants by electrode-neural coupling and neural regeneration
Source: Theranostics. 2026 Jan 1;16(2):936–51. doi: 10.7150/thno.120515 (PMC12675008; doi:10.7150/thno.120515)
Supplement: Supplementary file 1 — Supplementary figures. [file thnov16p0936s1.pdf]

Supplementary material

Neural stem cell-loaded biohybrid hydrogel improves cochlear implants by electrode-neural coupling and neural regeneration

Menghui Liao[#], Xin Zhou[#], Hao Wei[#], Yanru Qi[#], Pan Feng, Xin Gao, Yangnan Hu, Yuyang Qiu, Yusong Wang, Hongbo Yang[*], Zhonghong Zhang[*], Zhongze Gu[*], Reniie Chai[*]

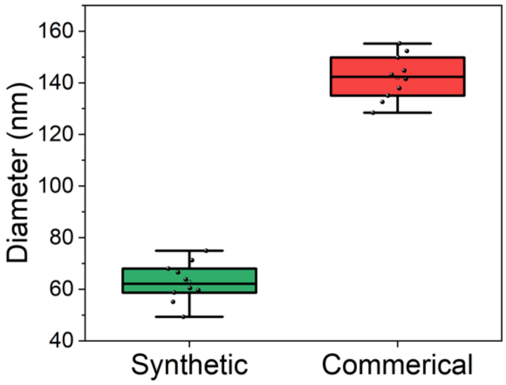

Figure S1. Particle size of our synthetic PEDOT:PSS and commercially available PEDOT:PSS.

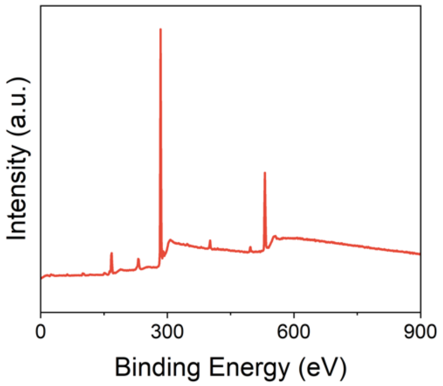

Figure S2. XPS pattern of PEDOT:PSS.

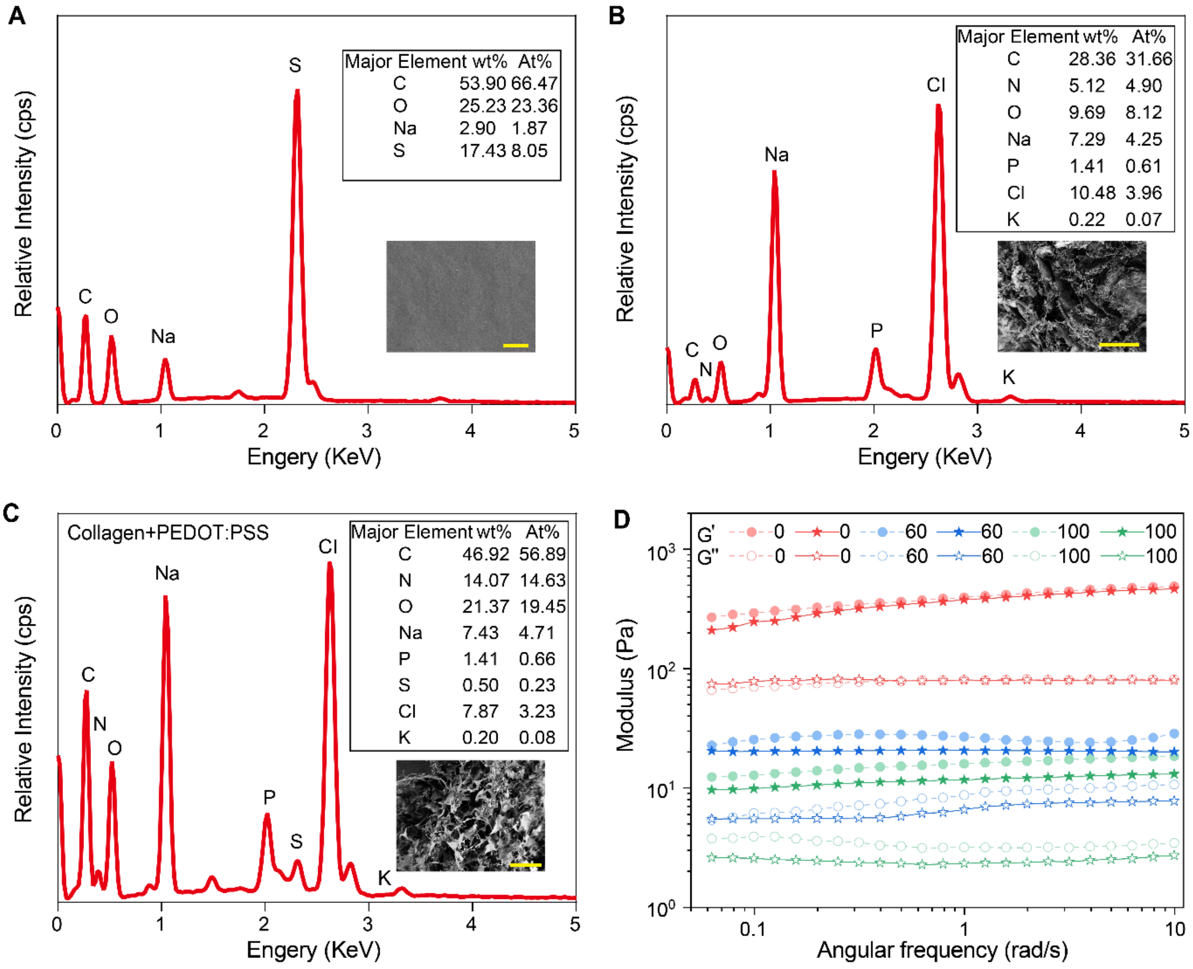

Figure S3. (A) SEM-EDS mapping of PEDOT:PSS. Scale bar = 10 µm. (B) SEM-EDS mapping of collagen hydrogel. Scale bar = 200 µm. (C) SEM-EDS mapping of PEDOT:PSS/collagen hydrogel. Scale bar = 200 µm. (D) Frequency-dependent rheological properties (storage modulus, G', and loss modulus, G'') of hydrogels with varying PEDOT:PSS content at 37 °C (Circle: hydrogels; star shape: NSC-loaded hydrogel).

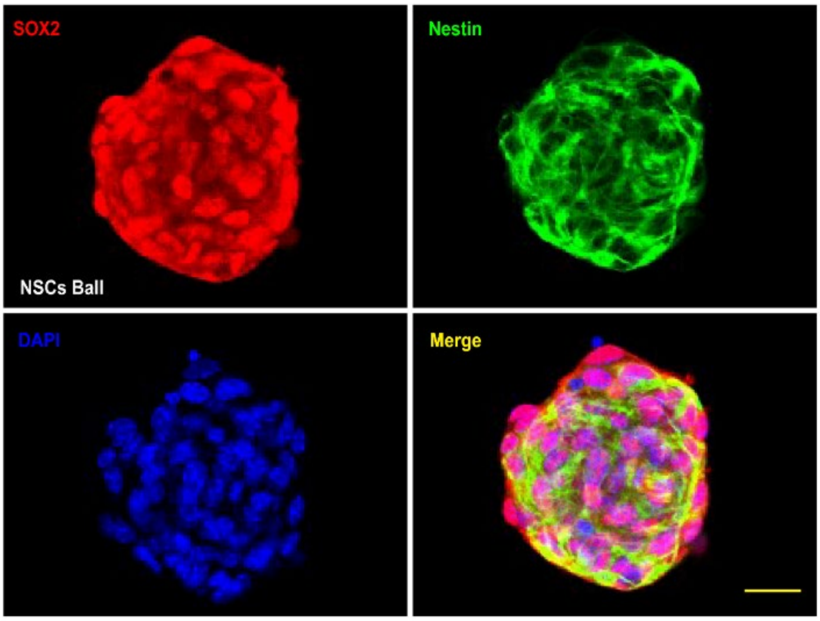

Figure S4. NSCs proliferate and form spheres *in vitro*. SOX2 marked NSCs (red), Nestin marked NSCs (green), and nuclei were stained by DAPI (blue). Scale bar = 20 μm.

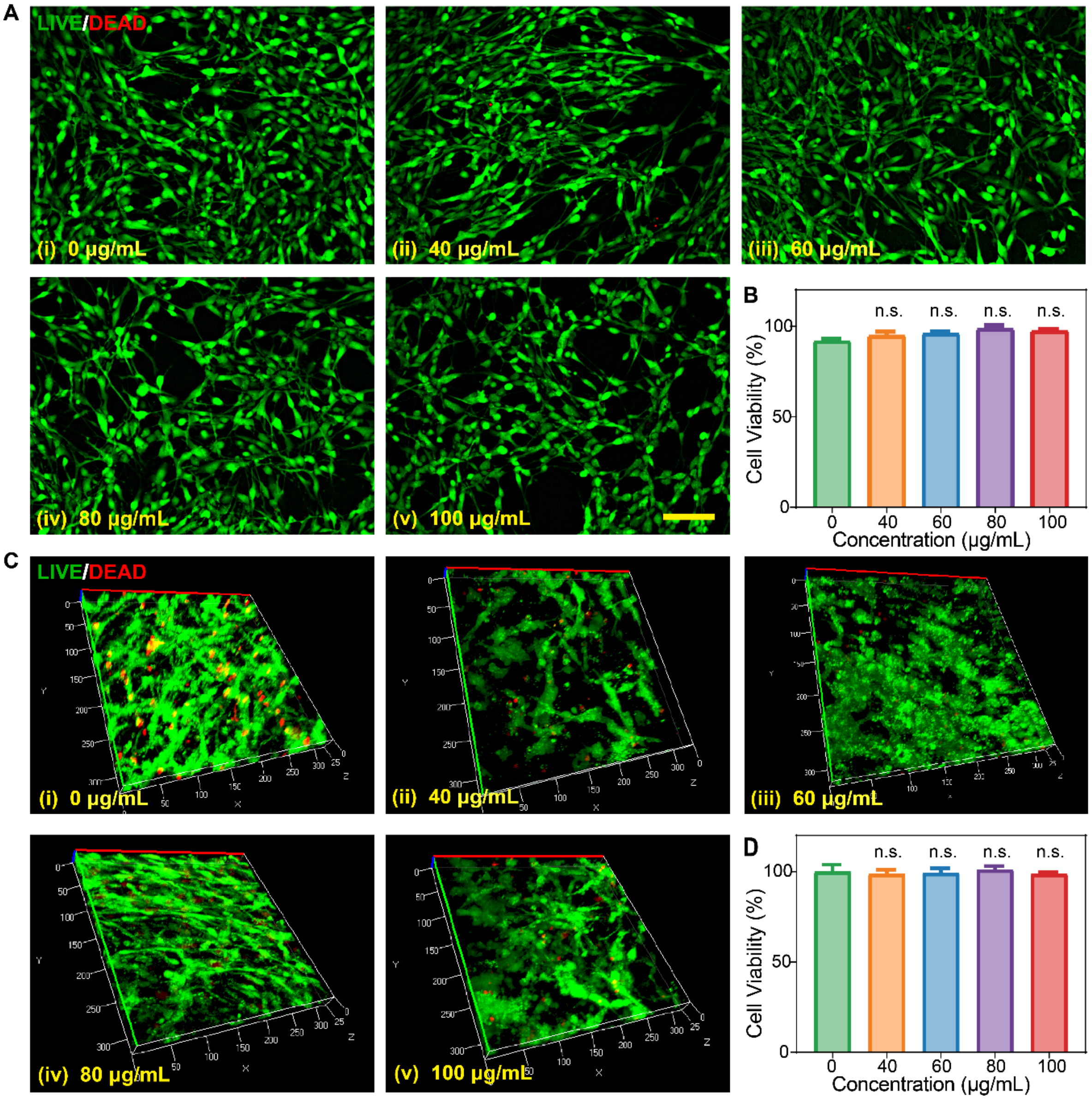

Figure S5. Biocompatibility evaluation of PEDOT:PSS and PEDOT:PSS/collagen hydrogel. (A) Representative live/dead fluorescence images of NSCs cultured in medium supplemented with PEDOT:PSS for 72 h. Live cells: Calcein-AM (green, cytoplasmic staining); dead cells: EthD-1 (red, nuclear staining). Scale bar = 100 μm. (B) CCK-8 assay quantifying NSC viability after 72-hour exposure to PEDOT:PSS in culture medium. (C) Live/dead staining of NSCs encapsulated in PEDOT:PSS/collagen hydrogels with varying PEDOT:PSS content after 5 days. Scale bar = 100 μm. (D) CCK-8 assay assessing NSC proliferation within hydrogels at day 7.

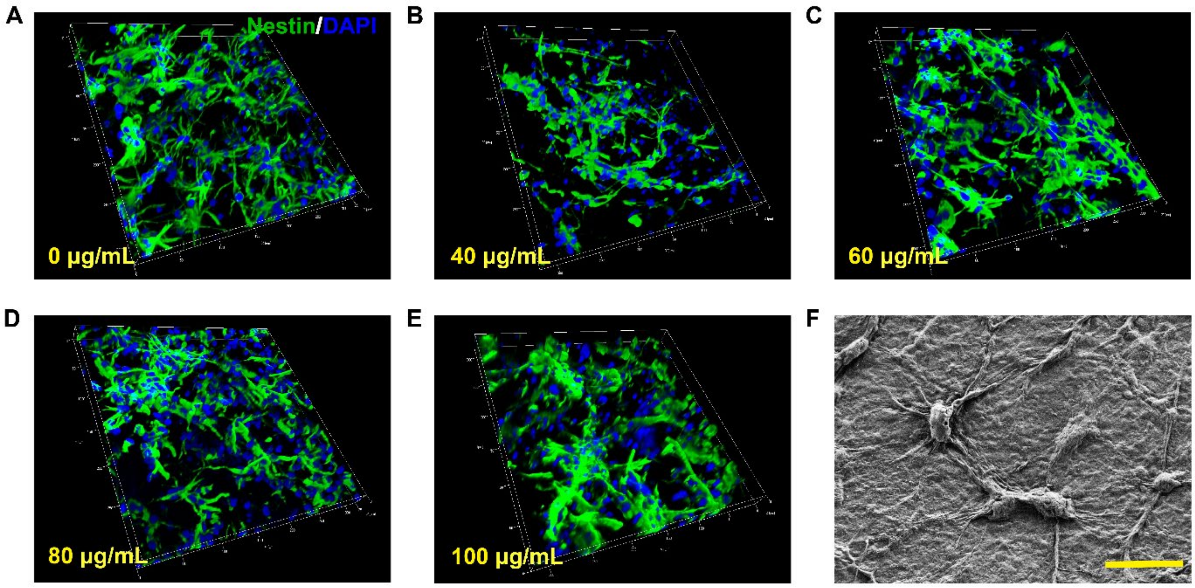

Figure S6. Adhesion and growth of NSCs in PEDOT:PSS/collagen hydrogels. (A–E) Fluorescence images of NSCs cultured for 72 hours in hydrogels with varying PEDOT:PSS content. Nestin (green, cytoskeletal marker for NSCs) and DAPI (blue, nuclei). Scale bar = 100 μm. (F) SEM image of NSCs cultured in PEDOT:PSS/collagen hydrogel for 3 days, demonstrating cell adhesion and interaction with the hydrogel matrix. Scale bar = 100 μm.

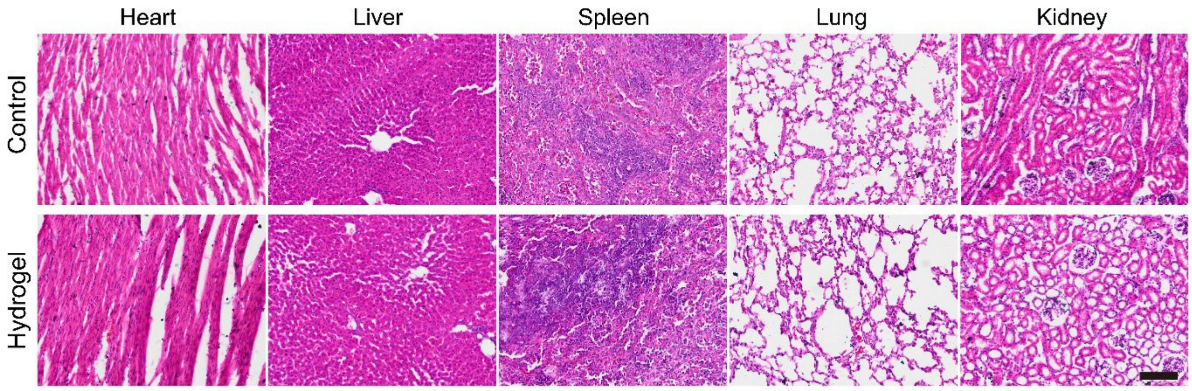

Figure S7. Toxicity evaluation *in vivo*. H&E staining of guinea pigs after 14 days treatments. Scale bar = 100 μm.

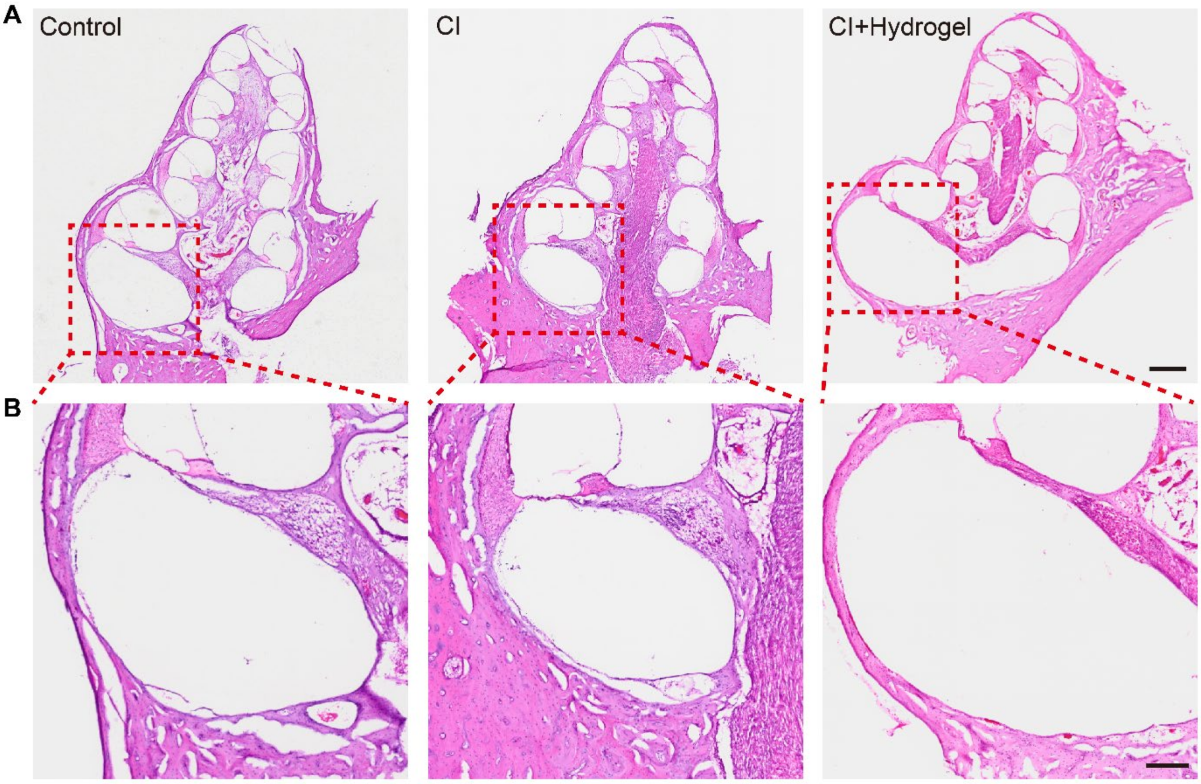

Figure S8. Cochlear H&E Staining Showed Normal Tissue Architecture. (A) Representative bright-field image of a whole cochlear cross-section (Scale bar = 500 μm). (B) Higher-magnification view of the boxed region in A showing detailed cochlear architecture (Scale bar = 200 μm).

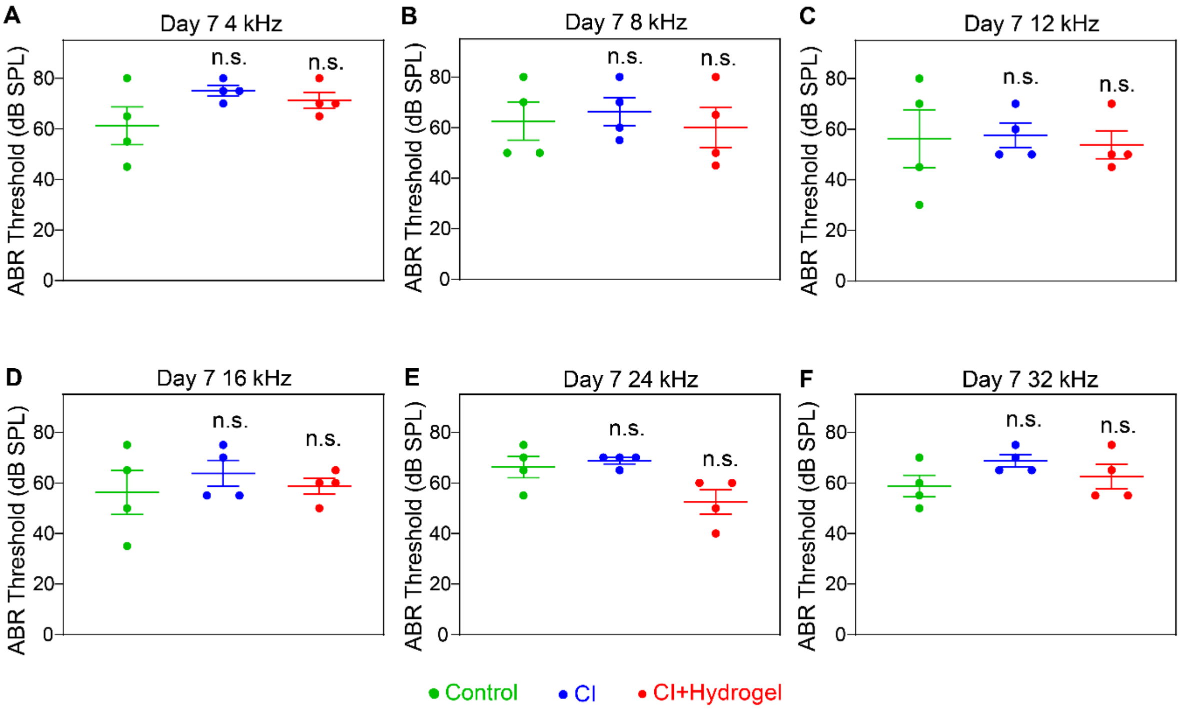

Figure S9. Functional hearing recovery in guinea pigs following CI surgery combined with NSC-loaded PEDOT:PSS/collagen hydrogel. Frequency-specific ABR thresholds measured at 7 days post-surgery for 4 kHz (A), 8 kHz (B), 12 kHz (C), 16 kHz (D), 24 kHz (E), and 32 kHz (F).
